# Phenotypic Characterization of Oral *Mucor* Species from Eurasian Vultures: Pathogenic Potential and Antimicrobial Ability

**DOI:** 10.3390/life13081638

**Published:** 2023-07-27

**Authors:** Catarina Raposo, Isa Serrano, Eva Cunha, Maria Patrícia Couto, Filipa Lopes, María Casero, Luís Tavares, Manuela Oliveira

**Affiliations:** 1 CIISA—Center for Interdisciplinary Research in Animal Health, Faculty of Veterinary Medicine, University of Lisbon, Avenida da Universidade Técnica, 1300-477 Lisboa, Portugal; catarina.raposo98@gmail.com (C.R.); evacunha@fmv.ulisboa.pt (E.C.); mp-couto@hotmail.com (M.P.C.); ltavares@fmv.ulisboa.pt (L.T.); moliveira@fmv.ulisboa.pt (M.O.); 2 Associate Laboratory for Animal and Veterinary Sciences (AL4AnimalS), 1300-477 Lisboa, Portugal; 3 CERAS—Centro de Estudos e Recuperação de Animais Selvagens, Quinta da Senhora de Mércules, 6000-909 Castelo Branco, Portugal; ana.f.lopes@cm-lisboa.pt; 4 RIAS Centro de Recuperação e Investigação de Animais Selvagens, Rua do Parque Natural da Ria Formosa, Quelfes, 8700-194 Olhão, Portugal; rias.aldeia@gmail.com

**Keywords:** *Aegypius monachus*, antimicrobial activity, rehabilitation centers, Eurasian vultures, *Gyps fulvus*, *Mucor* spp., virulence factors

## Abstract

Due to poisoning and decline in the food resources of Eurasian vultures, there has been a rise in the number of Griffon (*Gyps fulvus*) and Cinereous vultures (*Aegypius monachus*) needing veterinary care. In captivity, vultures often develop oral and other infectious diseases which can affect their survival and the probability of reintroduction in the wild. Therefore, it is important to characterize relevant microbial species present in the oral cavity of vultures, such as *Mucor* spp. In this work, seven *Mucor* spp. isolates previously obtained from *Gyps fulvus* and *Aegypius monachus* oral swabs collected at two rehabilitation centers in Portugal were characterized regarding their pathogenic enzymatic profile and antimicrobial activity. Isolates were identified by macro and microscopic observation, and PCR and ITS sequencing. Their antimicrobial activity was determined using a collection of pathogenic bacteria and two yeast species. Results showed that 86% of the isolates produced α-hemolysis, 71% expressed DNase, 57% produce lecithinase and lipase, 29% expressed gelatinase, and 29% were biofilm producers. Four isolates showed inhibitory activity against relevant human and veterinary clinical isolates, including *Escherichia coli*, *Enterococcus faecium*, *Neisseria zoodegmatis*, and *Staphylococcus aureus*. In conclusion, accurate management programs should consider the benefits and disadvantages of *Mucor* spp. presence in the oral mucosa.

## 1. Introduction

The existing 23 species of vultures worldwide are specialized scavengers which inhabit rugged and mountainous areas in Europe, Asia, Africa, and America, being extremely important for our ecosystem [1]. Vultures regulate the spreading of pathogenic agents and diseases and contribute to nutrient recycling by removing from the environment animal carcasses before putrefaction [2,3]. Vulture species have undergone dramatic declines in Europe, leading to the extinction of populations in the Alps and the Carpathians across central Europe. Despite this dramatic decline in some European vulture populations, Iberian populations are stable or even increasing due to conservation actions, and play a key role in the future viability of European vulture populations [4]. Currently, 61% of vulture species are threatened with extinction worldwide, and the most rapid declines have occurred in Asia and Africa [1,5]. Old World vultures are the most threatened, with over 80% of species in decline and 75% of species listed as Endangered or Critically Endangered [5]. This serious and long-term reduction is mainly due to illegal poisoning of carcasses of large mammals, either intentionally or unintentionally, decreased carrion supply arising from changes in livestock management practices and health regulations [6], contamination of carrion with veterinary drugs [4,7,8], and the environmental presence of toxic elements such as heavy metals and anticoagulant rodenticides [9,10]. That is, vulture decline was driven by humans [2,11], and it could severely impair their relevant role in ecosystems, from which humans deeply benefit [2].

In Portugal, the *Gyps fulvus* vulture is considered a Near-Threatened species [12], meaning that “it does not qualify for Critically Endangered, Endangered or Vulnerable now, but is close to qualifying for or is likely to qualify for a threatened category in the near future”[13], and worldwide they are classified as Least Concerned [13]. *Aegypius monachus* vultures are considered Critically Endangered in Portugal [12], and Near Threatened globally [13]. According to BirdLife International [13], of the four European vultures (Egyptian, Bearded, Griffon, and Cinereous), Griffon vulture (*Gyps fulvus*) populations are now increasing in Europe, whereas Cinereous vultures (*Aegypius monachus*) show a slow to moderate decline [1,13].

The remnant scavenger populations are isolated and in urgent need of conservation action assisted by international cooperation and expert consultants [11]. The number of vultures that are admitted to the wildlife rehabilitation and recovery centers in some European protected areas is high [14]. In the recovery centers, the recuperation rate is influenced by several factors, including the debilitation of each individual and the development of infectious diseases such as oral fungal infections [15].

Fungal infections pose a major threat, as mycoses are among the most frequent and serious systemic diseases in birds. The majority of fungal infections are caused by ubiquitous microorganisms that birds are continually exposed to [16]. These opportunistic mycoses could therefore have a major impact on populations because they may promote fledgling emaciation and dehydration caused by pain during food swallowing, subsequent difficulty in eating, and eventual death by starvation. In addition, some of these mycoses can be life-threatening if they become invasive [15,17,18].

Stress appears to be a defying factor in the development of fungal infections, being associated with captivity, inadequate management, or treatments with antimicrobials for long periods of time [15,17,19]. In addition, physiological stress, in the breeding season, for example, or in captivity, may contribute to the onset of these infections. Wild birds subjected to stress and suffering from immunosuppressive or debilitating diseases, malnutrition, and unsanitary conditions, can develop oral mycosis, with clinical lesions being more frequent in animals in captivity and in the offspring than in free-range wild birds and adults, respectively [17,18]. However, a recent study reported an outbreak of thrush-like lesions in the oral cavity of wild nestling vultures due to the consumption of livestock carcasses exposed to veterinary antibiotics [17]. Opportunistic fungal infections in the oral mucosa are mainly caused by *Candida albicans* and *Aspergillus fumigatus*, but *Mucor* and *Cryptococcus* may also have a major role in oral infections [20].

The order Mucorales includes several saprophytic fungi associated with underlying diseases. *Mucor* is composed by filamentous fungi found in soil, plants, and decaying fruits and vegetables. It belongs to phylum Zygomycota, order Mucorales, and family Mucoraceae [21], and is known for being ubiquitous in nature. Though *Mucor* is usually not pathogenic, several species are among the various zygomycetes identified as causing mucormycosis, an opportunistic fungal infection transmitted through the inhalation of spores [22]. Some *Mucor* spp. have been reported as possible etiological agents of meningoencephalitis in birds [23]. Additionally, they are described as being responsible for infections in humans, cattle, and swine [24].

In spite of their potential pathogenic profile, *Mucor* have important biotechnological potential as they are able to produce several enzymes, such as amylases, lipases, pectinases, and proteases, with many applications in pharmacy and industry [25]. For example, *Mucor circinelloides* enzymes are used in the biodegradation of diesel oil hydrocarbons [26]. These enzymes are essential proteins from the organisms’ metabolism, but many of them also act as virulence factors, playing an important role in host infection [25].

Moreover, it was already shown that *Mucor* spp. may have antibacterial activity towards relevant Gram-negative bacteria, namely, *Klebsiella pneumoniae*, *Escherichia coli*, *Pseudomonas brassicacearu*, and *Aeromonas hydrophila*, and also against Gram-positive bacteria, such as *Bacillus cereus* and *Staphylococcus aureus* [27,28]. Furthermore, *Mucor* spp. may present antifungal activity [27,28].

Considering the biotechnological potential of *Mucor* spp., the aims of this study were to characterize the pathogenic enzymatic profile and to evaluate the antimicrobial activity of *Mucor* isolates previously obtained from oral samples of Eurasian vultures in captivity.

## 2. Materials and Methods

### 2.1. Mucor Isolates

*Mucor* isolates under study (*n* = 7) were previously obtained from oral samples collected from six *Gyps fulvus* and one *Aegypius monachus* at two rehabilitation centers (Centro de Estudos e Recuperação de Animais Selvagens, CERAS, Castelo Branco (*n* = 2), and Centro de Recuperação e Investigação de Animais Selvagens, RIAS, Olhão (*n* = 5)), where they were recovering from bad nutrition and debility [29] (Table 1).

Being a ubiquitous species, *Mucor* isolates were likely transmitted through food or the environment. Oral samples were previously collected with AMIES swabs and transported to the Laboratory of Mycology of the Faculty of Veterinary Medicine, University of Lisbon, Portugal (FMV-ULisboa) [29]. The samples were cultured in Sabouraud Dextrose (SD) Agar and incubated for 5 days at 27 °C. After incubation, it was possible to presumptively identify seven isolates as *Mucor* spp. through macroscopic and microscopic evaluation, which were selected for this study. Isolates were maintained in SD agar at room temperature throughout the assays [30].

### 2.2. DNA Extraction

DNA extraction was carried out using the kit NucleoSpin Plant II by Macherey-Nagel, following the manufacturer’s instructions. First, the mycelium was washed and mixed in ethanol. Then, after removing the ethanol, the sample was placed in a reaction tube and siliconized glass beads were added along in 200 µL of lysis buffer. After homogenization, chloroform was added, and the sample was centrifuged for 5 min at 11,000 rpm. The supernatant was kept in a centrifuge tube and incubated at 65 °C for 30 min and, after another centrifugation for 2 min, it was collected using the NucleoSpin Filter. Again, the supernatant was placed in a reaction tube and the filter discarded. Afterwards, 450 µL of binding buffer were added and mixed, and the sample was put in a new collecting tube (NucleoSpin Plant II). Then, another centrifugation at 11,000 rpm was carried out for 1 min and the flow-through discarded. After this step, 400 µL of wash buffer were added to the NucleoSpin column, the sample was centrifuged again for 1 min, and the flow-through was discarded. Next, 700 µL of the second wash buffer were placed in the column, and another centrifugation was performed for 2 min to remove the buffer and allow the silica membrane to dry. The column was placed in another centrifuge tube together with 50 µL of the elution buffer, previously heated to 70 °C, and further incubated at 70 °C for 5 min. Lastly, the sample was centrifuged for 1 min to elute the DNA.

### 2.3. DNA Amplification and Sequencing

PCR was carried in a final volume of 25 µL, consisting of 0.4 µL (0.8 µM) of each primer [ITS1 (5′-TCC GTA GGT GAA CCT GCG G) and ITS2 (5′-GCT GCG TTC TTC ATC GAT GC)] [31], 10 µL of DNA, 10 µL of MasterMix (NZYTaq 2× Green, NZYTech^®^, Lisboa, Portugal) consisting in 1× reaction buffer (50 mM Tris–HCl, pH 9.0, 50 mM NaCl, 2.5 mM MgCl_2_, 200 µM each of dATP, dCTP, dGTP, dTTP), and 4.2 µL of PCR water. Amplification was performed according to the protocol described by Lau et al. [32] on a Doppio thermocycler (VWR^®^, Darmstadt, Germany). The conditions applied were: 95 °C for 10 min, followed by 60 cycles of 94 °C for 15 s, 55 °C for 30 s and 72 °C for 30 s, and a final extension at 72 °C for 5 min. Afterwards, to confirm amplification of fungal DNA, PCR products were separated by 1.5% agarose gel electrophoresis stained with Green Safe (NZYTech^®^), and results visualized by transillumination (ChemiDoc XRS+, Biorad^®^, Hercules, CA, USA). Then, PCR products were evaluated in Nanodrop to confirm their purity (ratio between absorbance at 260 nm and at 280 nm of ~1.8) and sent to Stabvida (https://www.stabvida.com/pt, accessed on 3 April 2023) for Sanger sequencing, in order to confirm the identification of isolates.

### 2.4. Phenotypic Characterization of Isolates Pathogenic Potential

To evaluate the virulence potential of the *Mucor* isolates under study, several tests were performed to assess the production of enzymes such as lipase, lecithinase, gelatinase, DNase, and hemolysins. Biofilm production ability was also tested. All assays were monitored after 24, 48, and 72 h incubation. The tests were conducted in triplicate on three independent days.

Lipase production was evaluated using the lipase medium, containing 1% peptone, 5% sodium chloride, 0.01% calcium chloride, 1% tween 80 (AppliChem GmbII, Darmstadt, Germany) and 2% agar (Difco, Detroit, MI, USA). After isolates’ inoculation and incubation at 37 °C, a positive result was associated with the appearance of a clear halo zone of precipitation around the colony [33].

Lecithinase production was determined using tryptic soy agar (35 g/L) (VWR, Leuven, Belgium) supplemented with 10% egg yolk (VWR, Leuven, Belgium). Isolates were then inoculated in the medium and incubated at 37 °C, after which a white precipitate around the colonies indicated lecithinase production [34].

Nutrient gelatin stab method was performed for the detection of gelatinase production by the *Mucor* isolates. Five ml of nutrient gelatin medium (Oxoid, Hampshire, UK), containing peptone (5 g/L), beef extract (3 g/L), and gelatin (120 g/L) were placed in a test tube. Then, after isolates’ inoculation and incubation at 37 °C, tubes were refrigerated at 4 °C for 30 min and observed in order to detect gelatin liquefaction [33].

DNase production was evaluated using the DNase medium supplemented with toluidine blue reagent (0.1 g/L) (VWR, Leuven, Belgium). Isolates were inoculated in the medium and further incubated at 37 °C, with a positive result corresponding to the formation of pink halos around the colonies [35].

To test hemolysin production, Columbia agar plates supplemented with 5% sheep blood (COS) (BioMérieux, Marcy-l’Etoile, France) were used. The isolates were inoculated and incubated at 37 °C, and observed for the development of a green or brown halo around the colonies, indicative of α-hemolysis, or of a clear halo, indicative of β-hemolysis [36].

Biofilm production was determined using Red Congo Agar, composed of Brain Heart Infusion (BHI) broth (37 g/L) (VWR, Leuven, Belgium), agar (20 g/L) (VWR, Leuven, Belgium), saccharose (50 g/L), and congo red indicator (8 g/L) (Sigma Aldrich, St. Louis, MI, USA) [37]. Isolates were then inoculated in the medium and further incubated at 37 °C, with the formation of a black halo around the colonies revealing a strong biofilm-forming ability after 24 h, a dark red halo revealing a moderate biofilm-forming ability after 48 h, and a red halo revealing a weak biofilm-forming ability after 72 h [37].

Finally, the virulence index (V. index) (Equation (1)) values were determined for all isolates [38]:

V. index = no. positive virulence factors/no. virulence factors tested(1)

### 2.5. Antimicrobial Activity

The capacity of the *Mucor* spp. isolates under study to inhibit different bacteria and yeast species was evaluated against a collection of eight potentially pathogenic bacteria from the Laboratory of Microbiology and Immunology of FMV-ULisboa, belonging to five species: the Gram-negative *Escherichia coli*, *Neisseria zoodegmatis* and *Pseudomonas aeruginosa*, and the Gram-positive *Enterococcus faecium* and *Staphylococcus aureus*, as well as two yeast species (*Candida* spp. and *Rhodotorula* spp.), also previously isolated from the oral cavity of vultures [29,39,40] (Table 2).

The inhibitory capacity of excreted metabolites produced by the *Mucor* isolates against bacteria and yeast species was determined by a spot-on-lawn test [41,42].

First, each *Mucor* isolate was inoculated in three separate tubes containing SD broth and incubated at 27 °C for 24 h (tube 1), 48 h (tube 2) and 72 h (tube 3). By the end of each incubation period, fungal suspensions were filtered using 0.2 µm filters, in order to obtain a sterile filtrate containing the metabolites produced by the isolates under evaluation.

The bacteria mentioned in Table 2 were inoculated in BHI agar plates and incubated at 37 °C for 24 h, while the yeasts were inoculated in Sabouraud medium and incubated at 27 °C for 48 h. Then, microbial suspensions with a concentration of 1 × 10^7^ CFU/mL were prepared in sodium chloride, and inoculated on the surface of Mueller–Hinton agar plates in order to form a uniform lawn of microbial growth.

Afterwards, 10 µL of the sterile filtrate obtained from each *Mucor* isolate at 24 h, 48 h, and 72 h were spotted on the surface of each microbial lawn, after which plates were incubated for 24 h at 37 °C. Then, plates were observed for the development of inhibition halos in the spot where the *Mucor* filtrates were spotted.

Experiments were conducted as three independent assays.

## 3. Results

### 3.1. Macroscopic and Microscopic Identification of Mucor spp.

The seven *Mucor* spp. isolates (M1 to M7) previously obtained from oral samples collected from six *Gyps fulvus* (samples 1, 2, 3, 5, 6, 7) and one *Aegypius monachus* (sample 4), were first presumptively identified through their macro and microscopic features.

**Figure 1:**
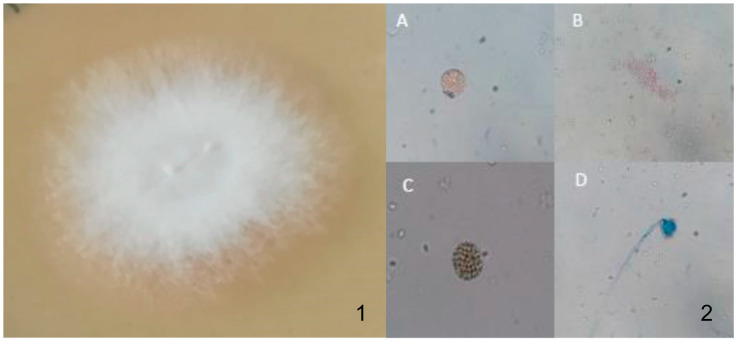
(**1**)—Macroscopic morphology of a *Mucor* colony; (**2**)—Microscopic characteristics of *Mucor* spp. (**A**)—Chlamydospore; (**B**)—Conidia; (**C**)—Sporangia; (**D**)—Sporangia, columella, and conidiophore. Amplification, 400×. Original.

Macroscopically, *Mucor* typically forms large white or beige colonies after 48 to 96 hours’ incubation, which can become grey or brownish due to the development of spores (Figure 1(1)).

Microscopically, the vegetative mycelium of *Mucor* isolates is non-septate or sparsely septate, and present broad (6–15 μm) hyphae without stolons and rhizoids, sporangiophores, sporangia, and spores. Some *Mucor* species produce chlamydospores (Figure 1(2A)), which are asexual spores, thick-walled and non-deciduous, well adapted to maintain viability through periods of dormancy [30,43]. *Mucor* spores or sporangiospores are round or elongated (Figure 1(2B)), and form apical, globular sporangia. These are round, grey to black in color, and filled with sporangiospores (Figure 1(2C)), and are supported and elevated by a well-defined column-shaped columella (Figure 1(2D)). Columellae are hyaline or dematiaceous and are hardly visible if the sporangium has not been ruptured [30,43].

### 3.2. Molecular Identification of Mucor spp.

**Figure 2:**
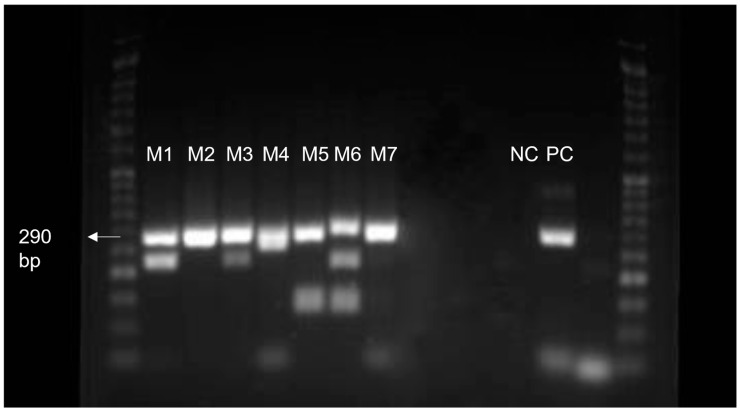
PCR products after separation by 1.5% agarose gel electrophoresis. M1 to M7, *Mucor* spp. isolates; NC, negative control; PC, positive control. NZYDNA ladder VI (NZYTech^®^) can be observed in the first and last wells. Original.

DNA was extracted from the *Mucor* spp. isolates under study for posterior amplification of the ITS region to allow their molecular identification. As shown in Figure 2, the seven DNA samples produced amplicons with the expected size, 290 bp [31,32] (Figure 2), concordant to *Mucor* spp.

Afterwards, PCR products were sent to Stabvida for Sanger sequencing. All isolates were identified as *Mucor* spp., with isolates M2, M3, M4, M5 and M7 being specifically identified as *Mucor circinelloides*.

### 3.3. Phenotypic Chacaterization of Mucor spp. Pathogenic Profile

The phenotypic production of enzymes by the *Mucor* spp. isolates, including lipase, lecithinase, gelatinase, DNase, and hemolysins, was evaluated, together with their biofilm-forming ability (Table 3).

Isolates’ phenotypic ability to produce lecithinase, gelatinase, and DNase increased with incubation time. At 24 h, none of the isolates were able to produce any of the virulence factors tested. At 48 h, all the isolates remained unable to produce gelatinase, but 28.8% (*n* = 2) were able to produce DNase, and 42.9% (*n* = 3) were able to produce lecithinase. At 72 h, two isolates (28.8%) were able to produce gelatinase, four (57.1%) were able to produce lecithinase, and five (71.4%) were able to express DNase.

On another end, isolates’ ability to produce lipase or α-hemolysins remained constant throughout the 72 h incubation period, with three isolates (42.9%) being able to produce lipase and six (85.7%) being able to express α-hemolysins.

Regarding biofilm production, it was possible to observe that only isolates M2 and M4 were able to produce biofilm (*n* = 2, 28.8%). Isolate M4 showed a strong positive result throughout the 72 h of incubation, whereas isolate M2 showed a weak positive result only after 72 h of incubation.

Regarding the V. index, M2, M3, and M6 showed the highest V. index (0.67); followed by M1, and M5 (0.5), and then M4, and M7 (0.33).

### 3.4. Antimicrobial Potential of the Mucor spp. Isolates

*Mucor* spp. filtrates were considered to have inhibitory activity against the bacteria and yeasts tested, when at 24 h, 48 h or 72 h, at least two of three replicates evaluated were able to promote microbial growth inhibition.

Overall, *Mucor* spp. filtrates had no inhibitory activity against the yeasts *Candida* spp. S2-1 and *Rhodotorula* spp. S2-2, nor against *P. aeruginosa* (413/18, ATCC 27853, and Z25.1), and *S. aureus* ATCC 29213.

The 24 h filtrates presented no inhibitory activity against any of the tested bacteria. Some of the 48 h filtrates were able to inhibit bacterial growth: M3-filtrate was able to inhibit *N. zoodegmatis* CCUG 52598T; M7-filtrate was able to inhibit *E. coli* ATCC 25922; and M2 and M4 -filtrate were able to inhibit *E. faecium* CCUG 36804. Regarding 72 h filtrates, M2-filtrate was able to inhibit *N. zoodegmatis* CCUG 52598T and *E. coli* ATCC 25922, M4-filtrate was able to inhibit *N. zoodegmatis* CCUG 52598T, and M7-filtrate was able to inhibit *S. aureus* Z25.2 (Table 4).

## 4. Discussion

In this study, hemolysin activity was the most frequent virulence factor expressed by the *Mucor* spp. isolates originated from the oral cavity of vultures, as only one isolate did not show signs of hemolysis. According to Nayak et al. [44], several different fungal genera are able to produce hemolysins. Although it is probable that these proteins may be involved in the regulation of fungal growth, the expression of hemolysins able to lyse host cells and other microorganisms could help provide a survival advantage for the fungal species, allowing them to compete with other microorganisms present in the same environment for available nutrients and resources [44].

The second most frequent virulence factor produced by the isolates under study was DNase, as the majority showed a DNase-positive result by the end of the assays. These results are compatible with the ones presented by Thompson and Eribo [45], in which DNase production was detected in all *Mucor* isolates tested. Unfortunately, studies regarding DNase production by fungi are sparse and little information is available until now. Nevertheless, it has been associated with the ability of a fungal phytopathogen, *Cochliobolus heterostrophus*, of escaping the action of extracellular maize DNA, which according to Park et al. [46], plays a critical role in cellular defense, being able to trap these fungi [46].

More than half of the isolates were able to produce lecithinase, which is in agreement with studies which have focused on several fungi, like *Candida albicans*, *Cryptococcus neoformans*, and *Aspergillus*, in which the production of lecithinase can be one of the most predominant enzymatic activities expressed [47]. Lecithinase, also called phospholipase C, is an enzyme that splits the phospholipid lecithin, one of the main components of cell membranes, to produce diglyceride and phosphorylcholine, causing toxicity. Additionally, it can cause hemolysis and membrane disruption, leading to cell lysis, hence playing a role in pathogenicity and contributing to fungal virulence [47,48].

It was expected that lipase production by the *Mucor* isolates was higher, as most isolates investigated in previous studies showed a high capacity to produce this enzyme [49,50]. In one of those studies, seven of 18 Mucorales species, three of which identified as *Mucor* spp., produced lipase [49]. Moreover, according to Alves et al. [44], among fifty-six *Mucor* isolates tested, 66% produced lipase [50]. In other studies, the properties of the lipase produced by *Mucor griseocyanus*, *Mucor miehei*, and by the new species *Mucor lipolyticus* Aac-0102 were also evaluated [51,52,53]. In the study herein, only 43% of the isolates were revealed to be able to produce lipase, a subclass of esterases found in all living organisms and used for their normal functioning, performing an important role in the processing of lipids, transport, and digestion for nutrient acquisition. Lipases have particular properties, such as specificity, tolerance to temperature and pH fluctuation, catalytic activity in organic solvents, and nontoxicity, which make them desirable for biotechnological applications [54]. Contribution of lipases to fungal virulence was suggested for several species of *Candida* [55,56] and other fungi [57], and was associated with their ability of adhesion to host cells and host tissues, initiation of several inflammatory processes by affecting immune cells, and self-defense mediated by lysing competing microbiota [55,57].

Only 29% of *Mucor* isolates could produce gelatinase. In a previous study, the production of this enzyme was detected in several yeasts and fungi isolated from different zones of the Antarctica sea, particularly in *Trichosporon pullulans* and in *Geomyces pannorum*, which revealed high levels of gelatinase production [58]. In another study, the gelatinase produced by *Penicillium chrysogenum*, *Aspergillus ustus*, *Aspergillus terreus*, and *Paecilomyces* spp. was described as playing a key role in biodegradation [59]. Gelatinase’s main function is to transform gelatin into smaller polypeptides, peptides, and amino acids that can cross the cell and be used by the fungi. It is also considered a virulence factor [33]. In a study with dermatophytes, it was shown that gelatinase production ability contributed to the capacity of these fungi to break down the substrate present in the patient’s skin [33].

Biofilms are dense, highly hydrated cell clusters that are irreversibly attached to a substratum, to an interface or to each other, and are embedded in a self-produced gelatinous matrix composed of extracellular polymeric substances [60]. Biofilm formation can protect fungal pathogens from the innate immune system of the host, and, as they are highly resistant to antifungals, are extremely difficult to eradicate [61,62]. Filamentation in fungi may be a requisite for robust biofilm development and virulence of fungal biofilms, as they often penetrate the substrates on which they grow [60]. According to Sardi et al. [62], biofilm production has been reported in yeasts and fungi such as *Candida albicans*, *Cryptococcus neoformans*, *Cryptococcus gattii*, *Rhodotorula* spp., *Aspergillus fumigatus*, *Malassezia pachydermatis*, *Histoplasma capsulatum*, *Paracoccidioides brasiliensis*, *Pneumocystis species*, *Coccidioides immitis*, *Fusarium* spp., *Saccharomyces cerevisiae*, *Trichosporon asahii*, Mucorales and Blastoschizomyces. Moreover, Singh et al. [60] showed that some zygomycetes, such as *Rhizopus oryzae*, *Lichtheimia corymbifera*, and *Rhizomucor pusillus*, produced robust, highly intertwined, filamentous, adherent structure-like biofilms. However, in the present study, the production of biofilm was not a common virulence factor expressed by the *Mucor* isolates, as only less than one third of them were found to be biofilm-producers.

Overall, and regarding the V. index, five *Mucor* isolates showed a worrying V. index (M1, M2, M3, M5, M6), being able to produce half or more than half of the virulence factors studied. This result is not surprising, since Mucorales express several known virulence factors that can contribute to their pathogenicity and immune evasion [63].

Previously, *Mucor* spp. was shown to have antibacterial activity towards bacteria and fungi [27,28]. Surprisingly, none of the *Mucor* filtrates had inhibitory activity against *P. aeruginosa*, nor yeast. A study aiming to determine the antimicrobial activity of different fungal genera showed that the fungi isolated from the rhizosphere of cultivated plants in Algeria, namely, *Alternaria*, *Aspergillus*, *Cladosporium*, *Curvularia*, *Fusarium*, *Mucor*, and *Penicillium*, had potential to inhibit *S. aureus*, *E. coli*, and *K. pneumoniae*, and also a lower inhibitory ability against *P. aeruginosa* [64]. The filtrates with relevant antibacterial activity were obtained from *Mucor circinelloides* M2, M3, M4, and M7. *M. circinelloides* is a common species whose predominant form is filamentous, although it can grow as a yeast under certain conditions [65]. The more susceptible bacterium was *N. zoodegmatis,* presumably due to the thinner cell walls of Gram-negative bacteria leading to more prone action of the fungus on the bacterial growth [27,28]. However, *P. aeruginosa*, also a Gram-negative, was not susceptible to the action of *Mucor* filtrates, probably due to the action of efflux pumps [66], the presence of which has not yet been described in *N. zoodegmatis*, to the best of the authors’ knowledge.

The filtrate with higher inhibitory activity was the one produced by the M2 isolate, which showed inhibition towards three bacterial species. M2 also presented a high V. index, being able to produce lecithinase, DNase, hemolysis, and biofilm (Table 3), which, in spite of being virulence factors, may also contribute to bacterial inhibition. M4 and M7 filtrates showed inhibitory activity against two bacterial species. The M4 isolate was also found to be a strong lipase and biofilm producer, while the M7 isolate was also able to express DNase and hemolysin. M3 showed inhibitory activity only towards *N. zoodegmatis* CCUG 52598T, and also a high V. index, being a lipase, lecithinase, DNase, and hemolysin producer.

These are interesting results, as they point to the possibility of *Mucor* spp. presence in the oral cavity of vultures possibly contributing to microorganism control. Conversely, the ability of *Mucor* to express virulence factors may suggest that these fungi could have an opportunistic impact on vultures’ oral health [17], and consequently delay vultures’ recovery. Therefore, further studies must be performed to confirm if *Mucor’s* presence in the oral mucosa of vultures results in antimicrobial activity *in vivo*.

## 5. Conclusions

The results obtained indicated that *Mucor* spp. isolated from Near-Threatened Eurasian Griffon vultures, *Gyps fulvus*, and from one Critically Endangered Cinereous Vulture, *Aegypius monachus*, have a high virulence capacity, as all isolates were able to produce at least one of the two virulence factors tested. Hemolysis activity, DNase production, and lecithinase were the most frequent virulence factors produced by the isolates under study. Additionally, four *Mucor* extracts presented inhibitory capacity towards one to three bacterial species, including relevant species such as *E. faecium* CCUG 36804, *N. zoodegmatis* CCUG 52598T, *E. coli* ATCC 25922, and *S. aureus* Z25.2.

Given the pathogenic profile of the tested fungi and that these vultures are endangered species, further studies should be developed to fully characterize the pathogenic potential of relevant microbial species present in the oral cavity of these animals. Although this fungus is apparently not a disease-causing agent, it can eventually be responsible for opportunistic infections. Therefore, accurate management programs should consider the benefits and the disadvantages of *Mucor* spp. presence in the oral mucosa of vultures.

## Figures and Tables

**Table 1:** Overview of the samples’ origin, including vultures’ species and rehabilitation center in which animals were recovering.

Sample of Origin	Vultures’ Species	Rehabilitation Center
1	*Gyps fulvus*	CERAS
2	*Gyps fulvus*	RIAS
3	*Gyps fulvus*	CERAS
4	*Aegypius monachus*	RIAS
5	*Gyps fulvus*	RIAS
6	*Gyps fulvus*	RIAS
7	*Gyps fulvus*	RIAS

CERAS, Centro de Estudos e Recuperação de Animais Selvagens; RIAS, Centro de Recuperação e Investigação de Animais Selvagens.

**Table 2:** Bacterial and yeast isolates used to test the inhibitory capacity of *Mucor* spp. and their origin.

Bacteria/Yeast Isolates	Origin
*Escherichia coli* ATCC 25922	Culture collection
*Enterococcus faecium* CCUG 36804	Culture collection
*Neisseria zoodegmatis* CCUG 52598T	Culture collection
*Pseudomonas aeruginosa* ATCC 27853	Culture collection
*Pseudomonas aeruginosa* 413/18	Isolated from an otitis from a dog
*Pseudomonas aeruginosa* Z25.1	Isolated from a patient with diabetic foot infection
*Staphylococcus aureus* ATCC 29213	Culture collection
*Staphylococcus aureus* Z25.2	Isolated from a patient with diabetic foot infection
*Candida* spp. S2-1	Isolated from the oral cavity of a vulture
*Rhodotorula* spp. S2-2	Isolated from the oral cavity of a vulture

**Table 3:** Phenotypic production of lipase, lecithinase, gelatinase, DNase, hemolysins, and biofilm, by the different *Mucor* spp. isolates under study, after 24, 48 and 72 h incubation (results from three independent experiments).

*Mucor* spp. Isolate	Lipase			Lecithinase			Gelatinase			DNase			Hemolysin			Biofilm		
	24 h	48 h	72 h	24 h	48 h	72 h	24 h	48 h	72 h	24 h	48 h	72 h	24 h	48 h	72 h	24 h	48 h	72 h
M1	−	−	−	−	+	+	−	−	+	−	−	−	α-hemolysis ^1^			−	−	−
M2	−	−	−	−	+	+	−	−	−	−	−	+	α-hemolysis ^1^			−	−	+
M3	+	+	+	−	−	+	−	−	−	−	+	+	α-hemolysis ^1^			−	−	−
M4	+	+	+	−	−	−	−	−	−	−	−	−	-			+	+	+
M5	−	−	−	−	+	+	−	−	−	−	−	+	α-hemolysis ^1^			−	−	−
M6	+	+	+	−	−	−	−	−	+	−	+	+	α-hemolysis ^1^			−	−	−
M7	−	−	−	−	−	−	−	−	−	−	−	+	α-hemolysis ^1^			−	−	−

^1^ The results were the same at 24 h, 48 h and 72 h. −, not detected; +, detected.

**Table 4:** Antibacterial inhibitory activity of *Mucor* spp. filtrates obtained after 48 h and 72 h incubations against bacteria (results from three independent experiments).

*Mucor* spp. Filtrate	48 h ^1^	72 h ^1^
M2	*E. faecium* CCUG 36804	*N. zoodegmatis* CCUG 52598T, *E. coli* ATCC 25922
M3	*N. zoodegmatis* CCUG 52598T	-
M4	*E. faecium* CCUG 36804	*N. zoodegmatis* CCUG 52598T
M7	*E. coli* ATCC 25922	*S. aureus* Z25.2

^1^ Inhibitory activity of bacterial growth demonstrated in at least two of three independent assays.

## Data Availability

The data presented in this study are available on request from the corresponding author. The data are not publicly available due to privacy reasons.
